# MaEIL4-MaMADS36-MaACS7 module transcriptionally regulates ethylene biosynthesis during banana fruit ripening

**DOI:** 10.1093/hr/uhae345

**Published:** 2024-12-16

**Authors:** Maoni Fu, Yunke Zheng, Jing Zhang, Chengju Deng, Jianbin Zhang, Caihong Jia, HongXia Miao, Jingyi Wang, Sijun Zheng, Zhiqiang Jin, Xinguo Li, Jianghui Xie, Juhua Liu

**Affiliations:** Banana Genetic Improvement Department, National Key Laboratory of Tropical Crop Breeding, Institute of Tropical Bioscience and Biotechnology & Sanya Research Institute, Chinese Academy of Tropical Agricultural Sciences, 4 Xueyuan Road, 571101 Haikou, China; Hainan Key Laboratory for Protection and Utilization of Tropical Bioresources, Hainan Institute for Tropical Agricultural Resources, Chinese Academy of Tropical Agricultural Sciences, 4 Xueyuan Road, 571101 Haikou, China; School of Tropical Agriculture and Forestry, Hainan University, 58 Renmin Road, 570228 Haikou, China; Banana Genetic Improvement Department, National Key Laboratory of Tropical Crop Breeding, Institute of Tropical Bioscience and Biotechnology & Sanya Research Institute, Chinese Academy of Tropical Agricultural Sciences, 4 Xueyuan Road, 571101 Haikou, China; Banana Genetic Improvement Department, National Key Laboratory of Tropical Crop Breeding, Institute of Tropical Bioscience and Biotechnology & Sanya Research Institute, Chinese Academy of Tropical Agricultural Sciences, 4 Xueyuan Road, 571101 Haikou, China; Hainan Key Laboratory for Protection and Utilization of Tropical Bioresources, Hainan Institute for Tropical Agricultural Resources, Chinese Academy of Tropical Agricultural Sciences, 4 Xueyuan Road, 571101 Haikou, China; Honghe Tropical Agriculture Institute of Yunnan, 661300 Hekou, China; Banana Genetic Improvement Department, National Key Laboratory of Tropical Crop Breeding, Institute of Tropical Bioscience and Biotechnology & Sanya Research Institute, Chinese Academy of Tropical Agricultural Sciences, 4 Xueyuan Road, 571101 Haikou, China; Hainan Key Laboratory for Protection and Utilization of Tropical Bioresources, Hainan Institute for Tropical Agricultural Resources, Chinese Academy of Tropical Agricultural Sciences, 4 Xueyuan Road, 571101 Haikou, China; Hainan Key Laboratory for Protection and Utilization of Tropical Bioresources, Hainan Institute for Tropical Agricultural Resources, Chinese Academy of Tropical Agricultural Sciences, 4 Xueyuan Road, 571101 Haikou, China; Banana Genetic Improvement Department, National Key Laboratory of Tropical Crop Breeding, Institute of Tropical Bioscience and Biotechnology & Sanya Research Institute, Chinese Academy of Tropical Agricultural Sciences, 4 Xueyuan Road, 571101 Haikou, China; Hainan Key Laboratory for Protection and Utilization of Tropical Bioresources, Hainan Institute for Tropical Agricultural Resources, Chinese Academy of Tropical Agricultural Sciences, 4 Xueyuan Road, 571101 Haikou, China; Banana Genetic Improvement Department, National Key Laboratory of Tropical Crop Breeding, Institute of Tropical Bioscience and Biotechnology & Sanya Research Institute, Chinese Academy of Tropical Agricultural Sciences, 4 Xueyuan Road, 571101 Haikou, China; Hainan Key Laboratory for Protection and Utilization of Tropical Bioresources, Hainan Institute for Tropical Agricultural Resources, Chinese Academy of Tropical Agricultural Sciences, 4 Xueyuan Road, 571101 Haikou, China; Agricultural Environment and Resources Institute, Yunnan Academy of Agricultural Sciences, Bioversity International, 2238 Beijing Road, 650205 Kunming, China; Banana Genetic Improvement Department, National Key Laboratory of Tropical Crop Breeding, Institute of Tropical Bioscience and Biotechnology & Sanya Research Institute, Chinese Academy of Tropical Agricultural Sciences, 4 Xueyuan Road, 571101 Haikou, China; School of Tropical Agriculture and Forestry, Hainan University, 58 Renmin Road, 570228 Haikou, China; Banana Genetic Improvement Department, National Key Laboratory of Tropical Crop Breeding, Institute of Tropical Bioscience and Biotechnology & Sanya Research Institute, Chinese Academy of Tropical Agricultural Sciences, 4 Xueyuan Road, 571101 Haikou, China; Banana Genetic Improvement Department, National Key Laboratory of Tropical Crop Breeding, Institute of Tropical Bioscience and Biotechnology & Sanya Research Institute, Chinese Academy of Tropical Agricultural Sciences, 4 Xueyuan Road, 571101 Haikou, China; Hainan Key Laboratory for Protection and Utilization of Tropical Bioresources, Hainan Institute for Tropical Agricultural Resources, Chinese Academy of Tropical Agricultural Sciences, 4 Xueyuan Road, 571101 Haikou, China

## Abstract

The present research examined the regulatory function of MaEIL4 in the ripening process of banana. The findings demonstrated that MaEIL4 is a transcription factor with activity in the nucleus. The transient modulation of MaEIL4 expression in banana fruit slices has been found to exert a significant impact on maturation, either enhancing or inhibiting its progression, as shown by phenotype and endogenous gene expression*. MaEIL4, MaMADS36*, and *MaACS7* were coexpressed in bananas. MaEIL4 interacted with both the MaMADS36 protein and the TGAA box of the *MaMADS36* promoter to activate its expression. Moreover, MaMADS36 bound to the C(A/T)rG box of the *MaACS7* promoter to regulate fruit ripening. The results have characterized the mechanism of MaMADS36’s response to upstream ethylene signals and established a new module, MaEIL4-MaMADS36-MaACS7, which transcriptionally regulates banana fruit ripening. This research has enhanced our comprehension of the pivotal function of MaMADS36 in controlling fruit maturation and thus suggests new strategies for fruit shelf life improvement and postharvest loss reduction.

## Introduction

Bananas (*Musa* spp.) are monocotyledonous plants in the Musaceae family and *Musa* genus [1]. Bananas are the second largest fruit in the world, producing 135 million tons per year (FAO 2024). Bananas are one of the nutritional fruits and staple foods worldwide [2]. The banana industry significantly contributes to economic and social advancement, serving as a vital component in regional development strategies.

The banana fruit ripening process involves a significant surge, and subsequent decline in ethylene biosynthesis characterizes the initial stages of respiration dynamics [3]. The 1-aminocyclopropane-1-carboxylic acid synthase (ACS), a pivotal enzyme in ethylene biosynthesis, holds a critical position within metabolic pathways [3–5]. We found that of the 11 *ACS* genes, *MaACS7* was the only upregulated gene and that exhibited high levels of expression during banana ripening [6]. Ectopically expressed *MaACS7* under its native promoter in tobacco is sufficient to generate ethylene [7]. Ethylene signaling holds a paramount importance in the regulation of banana fruit maturation [8, 9]. Ethylene signaling is rapidly transmitted downstream to ethylene-insensitive 3/EIN3-like (EIN3/EIL) and ethylene response factors (ERFs) through a cascade of amplification of the transcription activator EIN2 to regulate the expression of downstream transcription factors (TFs) or target genes and ultimately activate ethylene-related reactions [10–12]. In tomatoes, EIN3 exhibits regulatory functionality by specifically interacting with the promoter region of MCM1-AGAMOUS-DEFICIENS-SRF (MADS)-box gene, thereby influencing the process of fruit ripening [7]. In bananas, 17 *EIL* genes have been isolated but no further function identification has been performed [9, 13]. Recently, 13 *MaEIL* genes were isolated, among which *MaEIL9* was found to have a significant role in fruit maturity in tomatoes [14]. The function of EIN3 in blocking ethylene production was demonstrated by motif deletion. EIN3-NAC and MADS-type positive feedback loops operated to control fruit ripening [7]. However, the regulatory mechanism of EIN3 on MADS during postharvest banana fruit ripening remains unclear.

MADS-box genes comprise evolutionarily conserved TFs that exist in nearly all studied eukaryotes [15, 16]. The *TAGL1* exerts regulatory role in controlling maturity and quality in tomatoes [17, 18]. The indispensable role of two specific E-class (SEP3) MADS-box TFs, *MaMADS1* and *MaMADS2*, is crucial for fruit maturation [8, 19, 20]. MaMADS36 (Ma05_t18560.1), a TF belonging to the AG-like MADS-box family, was isolated from banana fruit in the year 2009. Its expression is promoted by external ethylene and mitigated by ethylene absorbent [21]. *MaMADS36* overexpression in red bananas greatly improved postharvest fruit ripening and quality trait elaboration but inhibited its expression, delaying fruit ripening. MaMADS36 constitutes a vital interactor within a protein network consisting of 74 fellow molecules, playing a pivotal role in modulating fruit maturation and quality elaboration. Among these target genes, *MaACS7* (Ma04_g35640.1) gained our attention because its expression pattern was highly consistent with that of *MaMADS36* [22]. However, the precise mechanism underlying that MaMADS36 senses exogenous ethylene signals to regulate banana maturation remains to be elucidated. Here, the function of MaEIL4 in postharvest banana fruit ripening was investigated, and a new module, MaEIL4-MaMADS36-MaACS7, which transcriptionally regulates banana fruit maturity, was established. These results offer a theoretical underpinning for improving fruit shelf life and fresh-keeping technology.

## Results

### Ripening characteristics of postharvest fruits and differential expression of the ethylene signal transduction factors MaEIN2 and MaEILs

**Figure 1:**
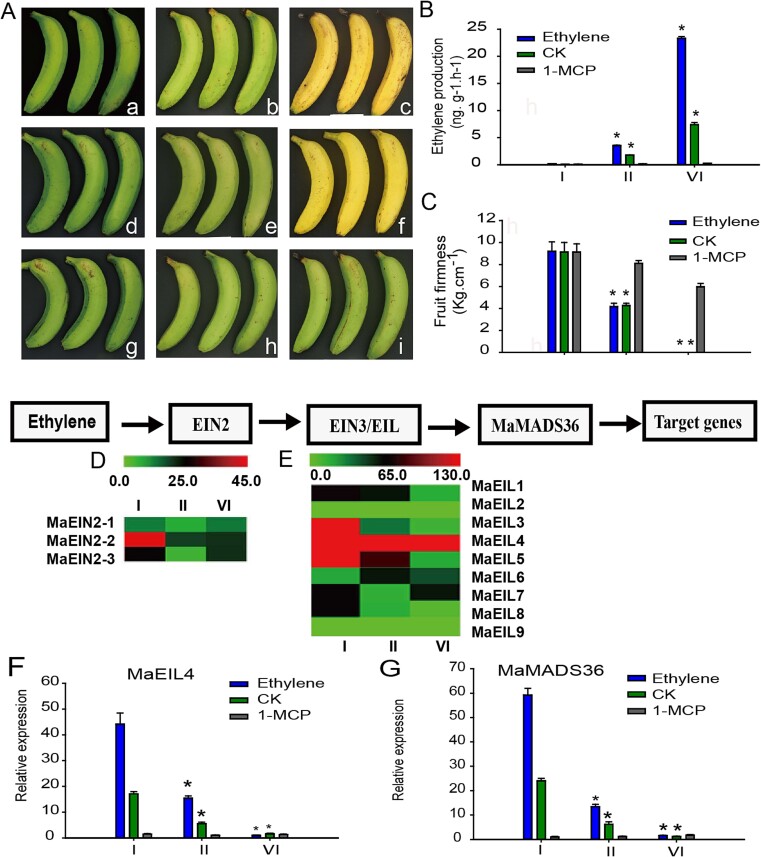
Ripening characteristics of postharvest banana fruits with different treatments and differential expression of ethylene signal transduction factors in banana fruit under different treatments. (A) Ripening characteristics of postharvest banana fruits under different treatments. a, b, and c represent the ethylene-treated fruits at ripening Stages I (0 DPH), II (2 DPH), and VI (6 DPH), respectively; d, e, and f represent the naturally ripened fruits at ripening Stages I (0 DPH), II (8 DPH), and VI (14 DPH), respectively; g, h, and i represent the 1-MCP-treated fruits at 0, 8, and 14 DPH, respectively. (B) Ethylene production of postharvest banana fruits under different treatments. (C) Fruit firmness of postharvest banana fruits under different treatments. (D) Differential expressions of MaEIN2 family members in naturally ripened fruits, as detected by transcriptome analysis. (E) Differential expression of MaEIL family members in naturally ripened fruits, as detected by transcriptome analysis. (F) Quantitative real-time polymerase chain reaction (qRT-PCR) identification of MaEIL4 in banana fruits under different treatments. (G) qRT-PCR identification of MaMADS36 in banana fruits under different treatments.

Postharvest fruits were subjected to exogenous ethylene, 1-methylcyclopropene (1-MCP), and natural ripening treatments. The ripening indicators of ethylene release, texture, and color showed that ripening was accelerated by 8 days following ethylene treatment and was significantly slowed following 1-MCP treatment (Fig. 1 a, b, c). The naturally ripened fruits at Stages I, II, and VI were selected for transcription sequencing and analysis of the differential expression of the ethylene signal transduction factors of MaEIN2 and MaEILs was investigated. Three genes of the MaEIN2 family and nine genes of the MaEILs family were differentially expressed, as shown in Fig. 1d, e and Table S1. Among these, *MaEIL4* was notable due to its high expression level at the initial stage of fruit maturation. The expression of *MaEIL4* under different treatments was investigated further. Exogenous ethylene significantly promoted *MaEIL4* and *MaMADS36* expression, whereas their application were counteracted by 1-MCP (Fig. 1f, g).

### Cloning and bioinformatic prediction of *MaEIL4*

The full-length sequence of *MaEIL4* (Ma06_t17470.1) comprising 4304 bp was isolated from banana fruits. The sequence contained a 2016-bp upstream intron, a 1908-bp single exon (coding sequence, CDS), and a 329-bp downstream intron (Fig. S1a). The exon encoded 636 amino acids. The number of negatively charged amino acids (Asp+Glu) was 81, and there were 70 positively charged amino acids (Arg + Lys). The protein formula was C3104H4837N883O970S34, with a molecular weight of 71.13 kD and an isoelectric point of 5.70. All amino acids were hydrophilic. There was a typical EIN3 conserved domain from 313 to 969 bp suggesting that *MaEIL4* belongs to the EIN3 family member (Fig. S1b). Subcellular localization prediction indicated that the probability of MaEIL4 being located in the nucleus was 73.9% (Fig. S1c), which was characterized by a TF.

### Characteristics of MaEIL4

**Figure 2:**
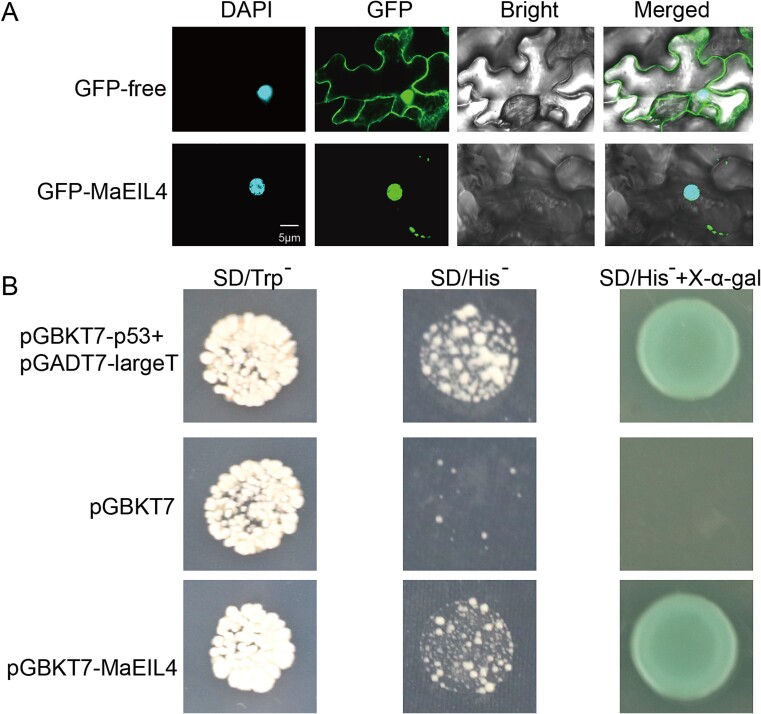
Characteristics of MaEIL4. (A) Subcellular localization. CK, GFP-free. (B) Transcriptional activity. CK^+^, pGBKT7-p53 + pGADT7-largeT.

For subcellular localization assay, the green fluorescent protein (GFP) used for the control (CK) was dispersed evenly throughout the cellular (Fig. 2a). However, GFP for the merged expression of pCAMBIA1300-MaEIL4-GFP was distributed in the cell nucleus. These results indicated that MaEIL4 was located in the nucleus, aligning with the prediction results. For transcriptional activity analysis, the positive control (CK^+^) of pGBKT7-p53 + pGADT7-largeT and pGBKT7-*MaEIL4* had blue plaques (Fig. 2b). However, the negative control of PGBKT7 did not turn blue. The findings demonstrated a transcriptional activation capacity for MaEIL4.

### Functional validation of MaEIL4 on fruit ripening

**Figure 3:**
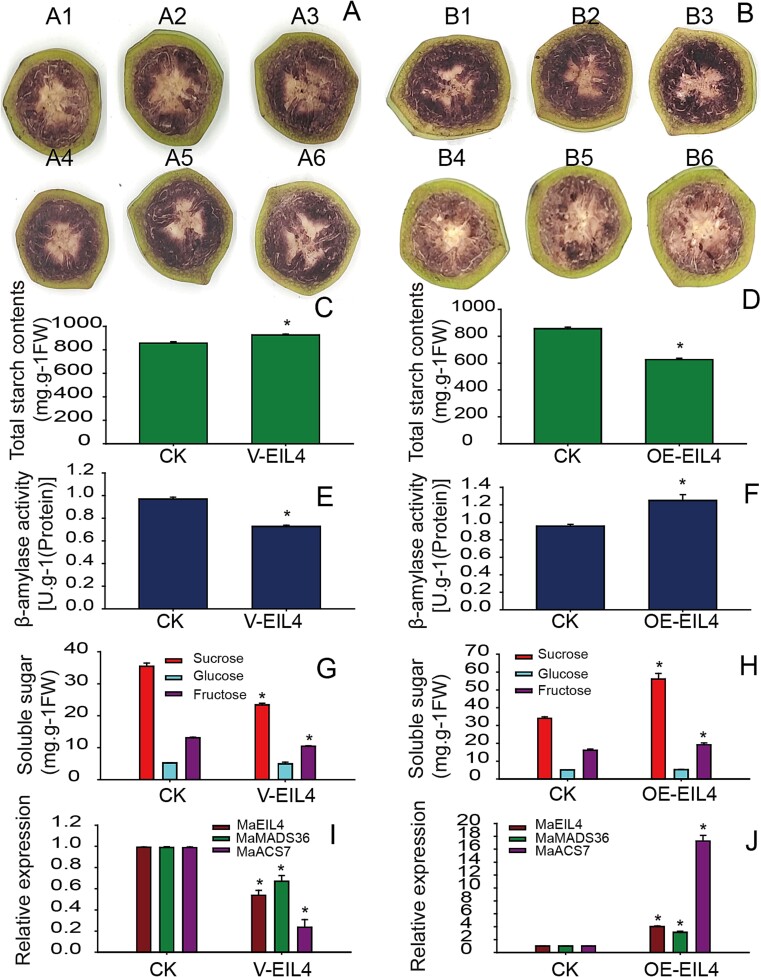
Function identification of MaEIL4 by VIGS and overexpression. (A) and (B) Effects of I_2_KI staining after *MaEIL4* transient VIGS and overexpression, respectively. (C) and (D) Total starch content after *MaEIL4* transient VIGS and overexpression, respectively. (E) and (F) β-amylase activities after *MaEIL4* transient VIGS and overexpression, respectively. (G) and (H) Soluble sugar content after *MaEIL4* transient VIGS and overexpression, respectively. (I) and (J) Endogenous gene expression of *MaEIL4*, *MaMADS36*, and *MaACS7* after *MaEIL4* transient VIGS and overexpression, respectively. CK, V-EIL4, and OE-EIL4 represent pTRV2-empty with pTRV1, pTRV2-*MaEIL4*, and pCAMBIA-*MaEIL4*, respectively. A1–A3 and A4–A6 represent three repeats for CK and V-EIL4, respectively. B1–B3 and B4–B6 represent three repeats for CK and OE-EIL4, respectively. The significance between groups was assessed by ANOVA (^*^  *P* < 0.05).

The role of MaEIL4 on fruit ripening traits was validated by transient virus-induced gene silencing (VIGS) and overexpression assays. The vectors of TRV2-*MaEIL4* and pCAMBIA1300-*MaEIL4* were constructed and transformed into fruit slices at 8 days postharvest (DPH). The results for iodine-potassium-iodide (I_2_KI) staining, the concentration of total starch and soluble sugar, β-amylase activity, and gene expression indicated that silencing *MaEIL4* generated a phenotype with suppressed starch degradation, fruit ripening, and quality trait elaboration (Fig. 3a, c, e, g, i). Conversely, *MaEIL4* overexpression generated a phenotype with improved starch degradation, fruit ripening, and quality trait elaboration (Fig. 3b, d, f, h, j).

### MaEIL4 interacted with MaMADS36

**Figure 4:**
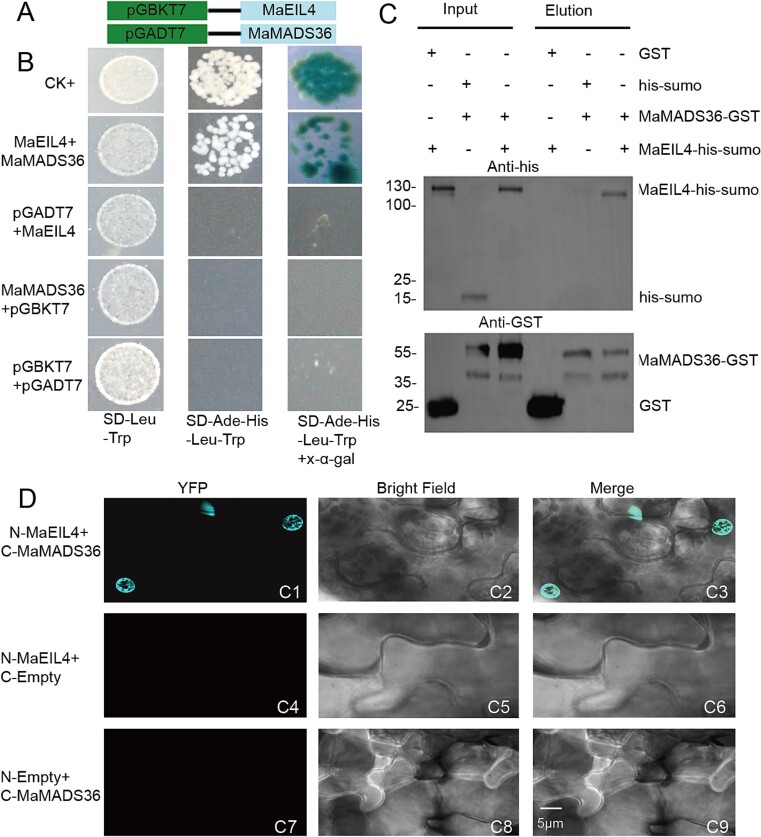
Interaction between MaEIL4 and MaMADS36. (A) Schematic diagram of vector construction for the Y2H assay. (B) Y2H assay. (C) Pull-down assay. (D) BiFC assay. CK^+^, pGBKT7–53 + pGADT7-T-antigen.

A yeast two-hybrid (Y2H) assay was employed to examine the interplay between MaEIL4 and MaMADS36. *MaEIL4* and *MaMADS36* were fused into pGBKT7 (BD) and pGADT7 (AD), respectively (Fig. 4a). pGBKT7-*MaEIL4* and pGADT7- *MaMADS36* constructs were successfully introduced into competent yeast strain AH109, followed by cultivation on selective media fortified with X-α-gal. Only for MaEIL4 and MaMADS36 coexpression and CK^+^, respectively, were blue clones observed. No blue clones were generated in the alternative combinations, specifically when AD was paired with MaEIL4 or MaMADS36 with BD, suggesting that MaEIL4 interacted with MaMADS36 (Fig. 4b). A GST pull-down assay was implemented to rigorously ascertain the interaction between MaEIL4 and MaMADS36. The results indicated that only GST-tagged MaMADS36 interacted with the His-sumo-tagged MaEIL4 protein (Fig. 4c). This interaction was further identified by bimolecular fluorescence complementation (BiFC). Only the integration of *MaEIL4*-pXY104 with *MaMADS36*-pXY106 generated fluorescence (Fig. 4d). The synergistic interaction between *MaEIL4*-pXY104 and *MaMADS36*-pXY106 was exclusively observed to elicit fluorescence.

### Bioinformatic analysis and activity detection of the *MaMADS36* promoter

**Figure 5:**
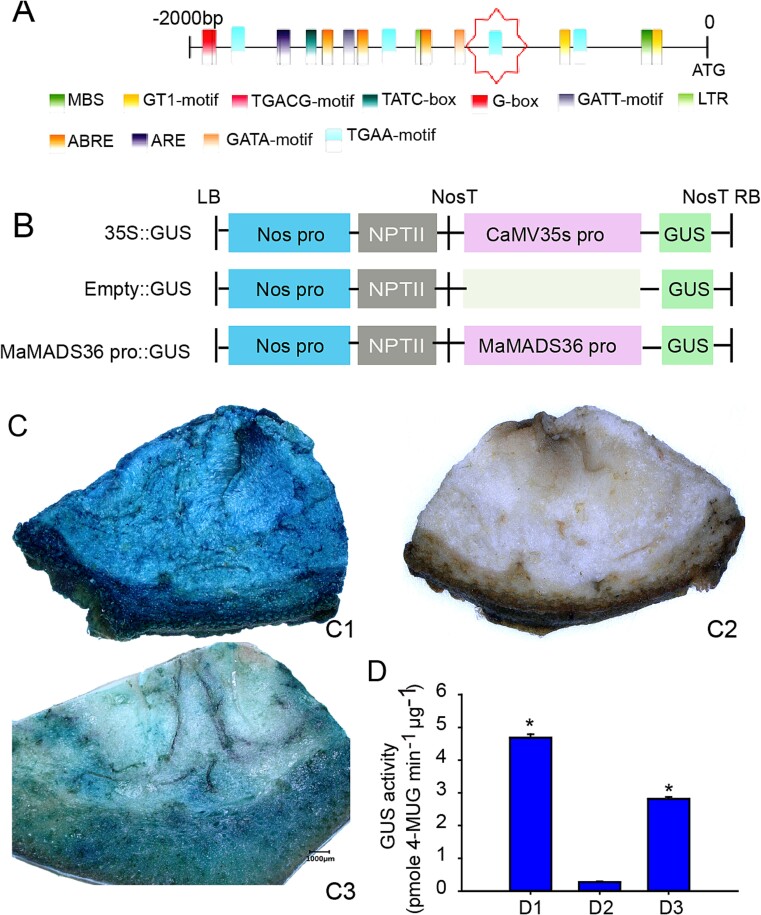
Analysis of MaMADS36 promoter activity. (A) Motif analysis of the MaMADS36 promoter. (B) Schematic diagram of vector construction. (C) GUS histochemical staining of banana fruit slices. C1, C2, and C3 represent the positive control (pBI121), the negative control (nonpromoter of pBI121), and MaMADS36 promoter, respectively. (D) GUS activity analysis. D1, D1, and D3 represent C1, C2, and C3, respectively. The significance between groups was assessed by ANOVA (^*^  *P* < 0.05).

The 2000-bp promoter of *MaMADS36* was amplified from banana fruits. There was an EIN3 binding site TGAA and 19 CAAT and nine TATAT boxes involved in initiating, promoting, and enhancing transcription. Promoter visualization was performed with TBtools [23] (Fig. 5a). The activity of the *MaMADS36* promoter was detected. The vectors constructed for the assay are shown in Fig. 5b. The pBI121 vector and its nonpromoter counterpart, designated as CK^+^ and CK^−^, respectively, were employed for the study. Promoter functionality was assessed through the visualization of β-glucuronidase (GUS) staining, where the former exhibited robust activity, manifesting in the deepest blue color, followed by pBI121-*MaMADS36*. CK^−^ did not show the blue color (Fig. 5c). The *MaMADS36* promoter’s GUS enzyme activity, as determined by GUS assay, exhibited a significant enhancement of 2.8-fold relative to CK^−^ but registered a 39.7% decrease compared to CK^+^ (Fig. 5d).

### Transcriptional regulation of MaEIL4 on *MaMADS36*

**Figure 6:**
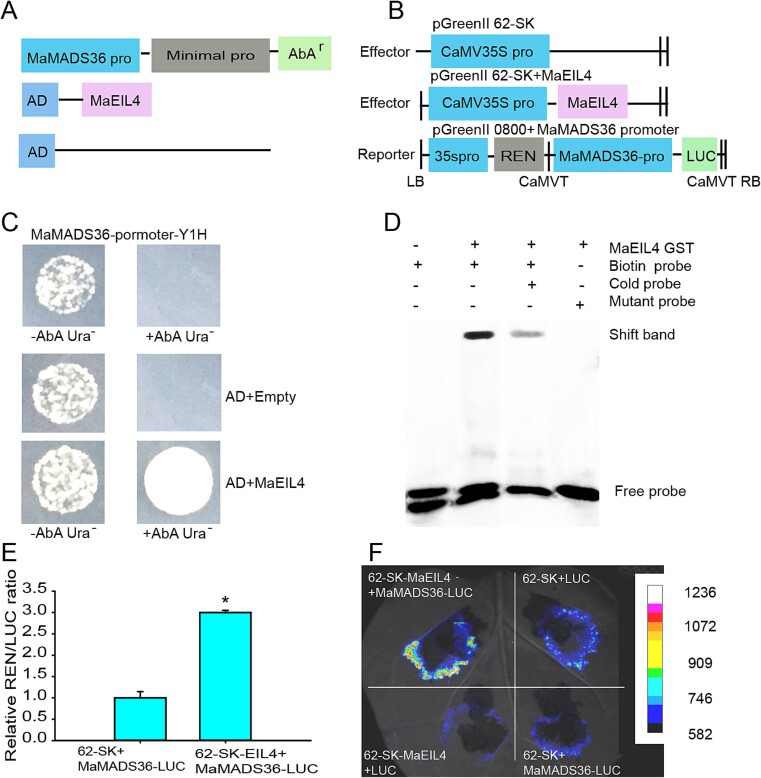
Transcriptional regulation of MaEIL4 on *MaMADS36*. (A) Schematic diagram of vector construction for the Y1H assay. (B) Schematic diagram of vector construction for LUC/REN assay. (C) Y1H assay. (D) EMSA. (E) Relative LUC/REN ratio. (F) Imaging detection *in vivo*.

The interaction mechanism of MaEIL4 with the *MaMADS36* promoter was investigated using a yeast one-hybrid (Y1H) assay. The vectors were constructed (Fig. 6a). Yeast cell proliferation was observed to be optimal under the influence of Aureobasidin A (AbA) with the cotransformation of the *MaMADS36* promoter and AD-*MaEIL4*. Conversely, transformation of yeast cells harboring the *MaMADS36* promoter did not yield growth upon coexpression with an empty AD (Fig. 6c), suggesting that MaEIL4 directly targets *MaMADS36*. Due to the discovery of the TGAA box within the *MaMADS36* promoter, an electrophoretic mobility shift assay (EMSA) employing purified recombinant MaEIL4 was conducted to investigate whether MaEIL4 exhibits direct binding affinity to the *MaMADS36* promoter *in vitro*. The recombinant MaEIL4 bound to the TGAA element of the *MaMADS36* promoter. Moreover, the unlabeled mutational probes couldn’t bind to MaEIL4 (Fig. 6d). Taken together, the results demonstrated that MaEIL4 specifically binds to the TGAA element of the *MaMADS36* promoter.

To delve deeper into the transcriptional regulation of MaEIL4 on MaMADS36, a dual-luciferase reporter (DLR) was employed. The vector construction is illustrated (Fig. 6b). The firefly luciferase/renilla luciferase (LUC/REN) ratio for *MaEIL4 +MaMADS36* exhibited a significant enhancement of 3.0-fold relative to the negative control (Fig. 6e). The findings indicated a potential interaction between MaEIL4 and the *MaMADS36* promoter, leading to enhanced luciferase expression. Similarly, the luciferase activity of *MaEIL4 + MaMADS36* exhibited a significant enhancement relative to the negative control (Fig. 6f), suggesting that MaEIL4 has transcriptional activation activity on the *MaMADS36* promoter.

### Transcriptional regulation of MaMADS36 on *MaACS7*

**Figure 7:**
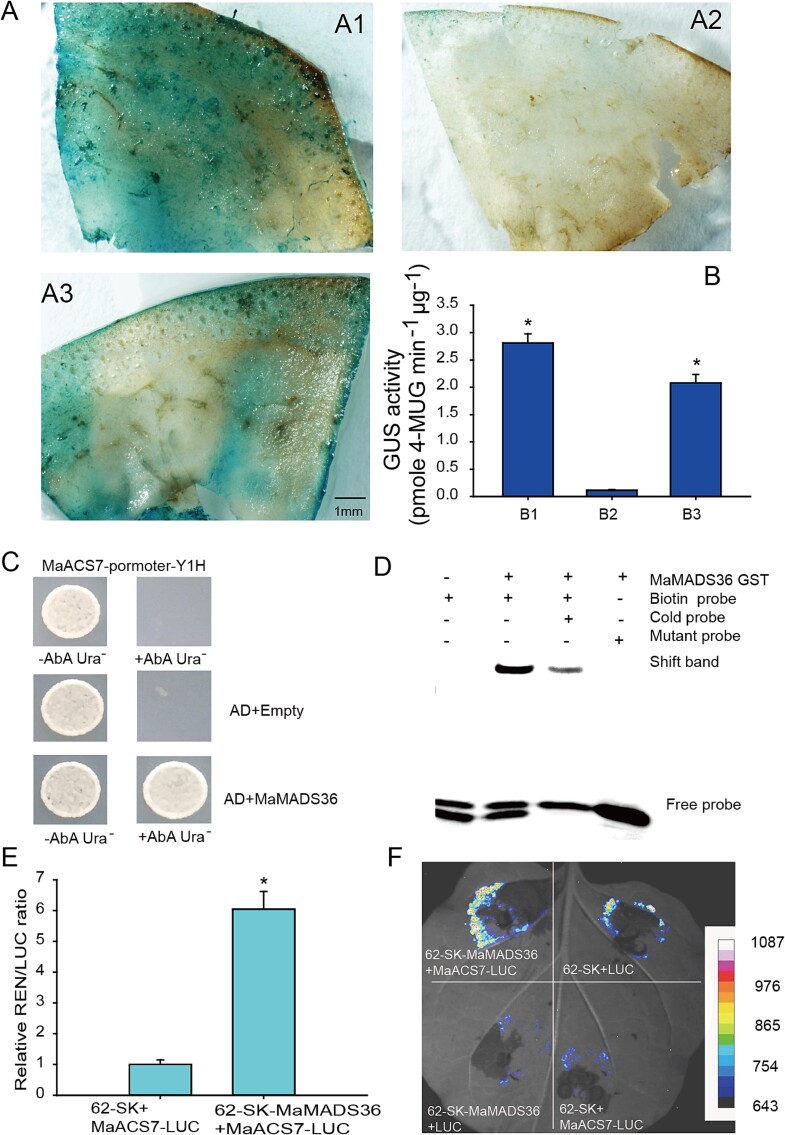
Transcriptional regulation of MaMADS36 on *MaACS7*. (A) GUS histochemical staining of banana fruit slices was used to analyze the activity of the *MaACS7* promoter. (B) GUS activity analysis. (C) Y1H assay. (D) EMSA identification. (E) Relative LUC/REN ratio. (F) Imaging detection *in vivo*. A1, A2, and A3 represent the positive control (pBI121), the negative control (nonpromoter of pBI121), and the *MaACS7* promoter, respectively. B1, B2, and B3 represent A1, A2, and A3, respectively. The differences between means were assessed by ANOVA (^*^  *P* < 0.05).

The *MaACS7* promoter was initially studied by bioinformatic analysis, and its activity was then detected. The 2000-bp promoter of *MaACS7* was amplified from banana fruits. There were 21 C(A/T)rG boxes responsible for MADS-box TF binding and 40 CAAT and 42 TATAT boxes responsible for initiating, promoting, and enhancing transcription. Promoter visualization was performed with TBtools [23] (Fig. S2). The pBI121 vector and its nonpromoter counterpart, designated as CK^+^ and CK^−^, respectively, were employed for the study. Promoter functionality was assessed through the visualization of GUS staining, where the former exhibited robust activity, manifesting in the deepest blue color, followed by pBI121-*MaACS7* (Fig. 7a). The *MaACS7* promoter’s GUS enzyme activity exhibited a significant enhancement of 21-fold relative to CK^−^ but registered a 25.0% decrease compared to CK^+^ (Fig. 7b). These results suggest that *MaACS7* has strong promoter activity.

The binding affinity between MaMADS36 and the MaACS7 promoter was examined through a Y1H assay. Yeast cell proliferation was observed to be optimal under the influence of AbA with the cotransformation of the *MaACS7* promoter and AD-*MaMADS36*. Conversely, transformation of yeast cells harboring the *MaACS7* promoter did not yield growth upon coexpression with an empty AD construct (Fig. 7c), suggesting that MaMADS36 targets *MaACS7*. Due to the discovery of 21 C(A/T)rG boxes within the *MaACS7* promoter, an EMSA employing purified recombinant MaMADS36 was conducted to investigate whether MaMADS36 exhibits direct binding affinity to the *MaACS7* promoter. The recombinant MaMADS36 bound to the C(A/T)rG element of the *MaACS7* promoter. Moreover, the unlabeled mutational probes could not bind to MaMADS36 (Fig. 7d). The collective findings indicated a specific interaction of MaMADS36 with the C(A/T)rG elements within the regulatory region of the *MaACS7*.

The transcriptional modulation of MaMADS36 in relation to *MaACS7* was deeply investigated by a DLR system. The comparative LUC/REN ratio of the construct pGreenII 62 SK-*MaMADS36* in conjunction with pGreenII 0800-*MaACS7* exhibited a statistically significant 6.0-fold enhancement over the control (Fig. 7e). The findings indicated a potential mechanism wherein MaMADS36 exerts its influence by directly binding to *MaACS7* promoter, thereby augmenting luciferase expression. Similarly, The luciferase expression in the composite construct pGreenII 62 SK-*MaMADS36* + pGreenII 0800-*MaACS7* exhibited a statistically significant enhancement compared to the control (Fig. 7f), strongly indicating that MaMADS36 has transcriptional activity on the *MaACS7* promoter.

## Discussion

### MaEIL4 responds to exogenous ethylene signals

Bananas are typical climacteric fruits, and ripening is primarily induced by exogenous ethylene. Therefore, ethylene biosynthesis and the signal transduction pathway play critical roles in postharvest banana ripening [3, 16]. We identified 26 ethylene signal transduction upstream factors in the banana fruits and five ethylene receptors (ETRs), nine constitutive triple response (CTRs), three EIN2, and nine EIN3/EILs that were differentially expressed during the banana ripening process based on transcriptome data and our previous research [6]. It represents the inaugural comprehensive investigation into the array of gene family constituents preceding the ethylene signaling cascade, and the number of genes involved was greatly expanded compared to the results of Jourda *et al*. [9]. Among these, *MaEIL4* was notable for its high expression level at the early stage of banana fruit ripening (Fig. 1e). *MaEIL4* expression exhibited a significant induction in response to ethylene and was effectively mitigated by 1-MCP (Fig. 1f), suggesting that MaEIL4 responds to exogenous ethylene signals in banana maturation phase. The obtained data demonstrated that *MaEIL4* gene expression is positively regulated by ethylene at the transcriptional level, in agreement with the reports of Mbéguié-A-Mbéguié *et al*. [13], Yan *et al*. [24], and Dolgikh *et al*. [25].

### Overexpression or silencing of *MaEIL4* in bananas improved or suppressed fruit ripening

Banana fruit slices are an ideal model system for investigating fruit ripening due to their ease of observation of the color changes after I_2_KI staining. These advantages facilitate the measurements of ripening and quality-related physiological indexes that have been widely used in investigating the roles of genes in banana fruit ripening [26–29]. We found that transient overexpression or silencing of *MaEIL4* in banana fruit slices significantly promoted or suppressed fruit ripening. Moreover, the expression levels of endogenous genes related to ethylene biosynthesis and fruit ripening, such as *MaEIL4*, *MaMADS36*, and *MaACS7* were also promoted or inhibited (Fig. 3), suggesting that MaEIL4 is an essential ethylene signal transduction factor that is crucial for banana maturation. The results were highly consistent with the findings of Liu *et al*. [15], Lü  *et al*. [7], and Zhu *et al*. [30].

### The MaEIL4-MaMADS36-MaACS7 module transcriptionally regulates banana maturity

The expression of MaMADS36, a factor that was previously isolated from banana fruits, was significantly influenced by exogenous ethylene. MaMADS36 also promoted endogenous ethylene biosynthesis [21]. These results indicated that MaMADS36 could not only sense upstream ethylene signal transduction factors but also interact with the key enzymes of ethylene biosynthesis, ultimately improving banana maturity. Here, we demonstrated that MaMADS36 could interact with MaEIL4 to form a protein–protein complex (Fig. 4). The findings align with those reported by Liu *et al*. [31, 32] and Zheng *et al*. [33] that MaMADS36 could bind with non-MADS-box TFs such as MuUBA1 and MaOFP1 to sense signals and coregulate banana fruit ripening. However, the MaMADS36 promoter was the target directly bound by MaEIL4 to form a complicated regulatory loop and transcriptionally regulate the expression of downstream genes (Fig. 6). These results have deepened and expanded the findings of Lü *et al*. [7], who reported that EIN3 could bind the MADS-box promoter to modulate fruit maturity in tomatoes. The latter results were highly consistent with the reports that the ethylene signal is rapidly transduced through a cascade of ethylene signal factors to govern the transcriptional regulation of downstream TFs or target genes [10, 12].

*MaACS7* was coexpressed with *MaMADS36* during the banana fruit maturity [6, 22]. Here, *MaACS7* had strong promoter activity due to multiple MADS-box TF-binding domains. Further investigation revealed that MaMADS36 directly bound with the C(A/T)rG box of the *MaACS7* promoter to transcriptionally regulate its expression (Fig. 6). This was consistent with the report by Choudhury *et al*. [34], who discovered that banana MADS-box TFs play a regulatory role in the ripening process by binding to the CArG-box of the *MA-ACS1* promoter.

## Conclusion

**Figure 8:**
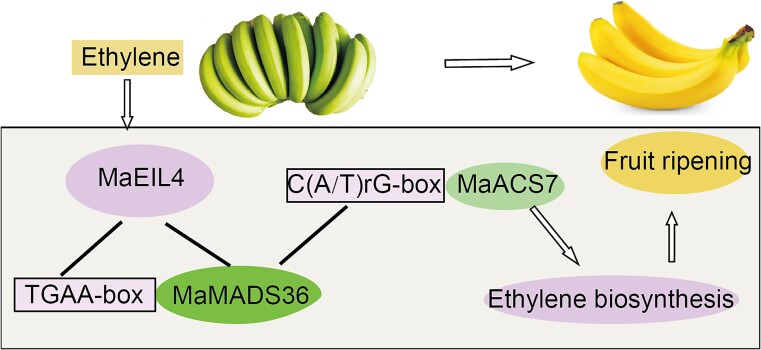
The MaEIL4-MaMADS36-MaACS7 module transcriptionally regulates banana fruit ripening.

We have demonstrated that MaEIL4 is a TF. The transient modulation of *MaEIL4* expression in banana fruit slices has been found to exert a significant impact on ripening processes, either enhancing or inhibiting its progression. MaEIL4 not only interacted with MaMADS36 but also bound with the TGAA box of the MaMADS36 promoter. Moreover, MaMADS36 exhibits a direct interaction with the C(A/T)rG-rich region of the *MaACS7* promoter to regulate fruit ripening (Fig. 8). These results have not only characterized the mechanism of MaMADS36 response to upstream ethylene signals but have also established a new module, MaEIL4-MaMADS36-MaACS7, which transcriptionally regulates ethylene biosynthesis during banana fruit ripening.

## Materials and methods

### Plant materials and treatments

Baxi (*Musa* spp. Cavendish) bananas were procured from our banana plantation. Healthy middle-fruit combs were sampled. Disinfect the fruits surface with 0.1% sodium hypochlorite for 10 min and air dry naturally. About 300 fruits were divided into three groups for different treatments with reference to Liu *et al*. [31]. The naturally ripened and exposed for 12 h to 100 μl l^−1^ ethylene-treated fruits at Stages I, II, and VI and the 1 μl l^−1^ 1-MCP-treated fruits corresponding to those days were sampled and quickly frozen in liquid nitrogen and stored at −80°C for later use. The 60 naturally ripened fruits slices, measuring 2–4 mm in thickness, were accurate segmentation at 8 DPH for VIGS and transient overexpression assays.

### Detection of fruit quality-related indicators

I_2_KI dyeing was done with reference to Liu MT *et al*. [26]. Ethylene release, firmness, total starch content, β-amylase activity, and soluble sugar content were determined with reference to Liu *et al*. [22].

### Transcriptome data

The transcriptome data for naturally ripened fruits at Stages I (0 DPH), II (8 DPH), and VI (14 DPH) were obtained from the NCBI-SRA database (accession number: PRJNA343716) [6].

### Quantitative real-time polymerase chain reaction

Total RNA was obtained from banana fruits using the RNAprep Pure kit (TIANGEN, A0711A). First-strand cDNA was synthesized according to the method of Liu *et al*. [22]. Expression detection of *MaEIL4*, *MaMADS36,* and *MaACS7* was operated according to the methods of Chen *et al*. [35]. The internal controls MaRPS2 (HQ853246) and MaUBQ2 (HQ853254) were introduced for the normalization of target gene expression.

### Subcellular localization and transcriptional activity assay

*MaEIL4* cDNA was attached to pCAMBIA1300 to form the plant expression vector pCAMBIA1300-MaEIL4-GFP using the digestion sites of X*ba*I and S*al*I and then transformed into Agrobacterium EHA105. A single clone was selected for activation; when the OD600 reached 0.8, the clone was injected into tobacco leaves. The injected leaves were placed under low light for 1–3 days at 25°C, and then the distributions of GFP and 4′,6-diamidino-2-phenylindole (DAPI) were examined under a Laser Capture Microdissection System (OLYMPUS FV10-SU FV1000, Japan).

To investigate transcriptional activity, a yeast expression vector pGBKT7-*MaEIL4* was constructed and transformed into Y2H Gold competent cells. The transformed cells grew on selective media fortified with X-α-gal.

### VIGS and overexpression assays

A 770-bp gene fragment of *MaEIL4* was isolated from banana cDNA using X*ba*I and K*pn*I restriction sites and was fused to pTRV2, obtaining pTRV2-*MaEIL4.* A 1905-bp gene fragment of *MaEIL4* was isolated using *Xba*l and *Sal*l restriction sites and was fused to pCAMBIA1300*.* GV3101 cell cultures carrying pCAMBIA1300-*MaEIL4*, pTRV2, and pTRV2-*MaEIL4* were combined with pTRV1 in a 1:3 ratio. The transformation was operated with reference to Miao *et al*. [27]. The banana slices were selected for I_2_KI staining, gene expression analysis, and quality-related indicator analysis.

### Y2H assay

*MaEIL4* was fused with pGBKT7. *MaMADS36* was fused with pGADT7. Self-activation was examined on selective media fortified with X-α-gal. The resulting products were cotransformed into the competent yeast cells. The interactions were assessed according to the method of Liu *et al*. [28].

### Pull-down assay

The full-length cDNA of *MaMADS36* was fused into pGEX-4 T-1, whereas *MaEIL4* was fused into pET-32a. The pull-down assay was performed according to the method of Zheng *et al*. [36].

### BiFC assay

The *MaEIL4* cDNA was cloned to pXY106 (nYFP), and *MaMADS36* was cloned to pXY104 (cYFP). The BiFC assay was operated according to the method of Zheng *et al*. [36].

### Promoter bioinformatic analysis

The *MaMADS36* and *MaACS7* promoters were obtained from banana genomic DNA. The promoter bioinformatic analysis was performed according to the method of Liu *et al*. [32].

### GUS detection

The 35S promoter of pBI121 was substituted by the *MaMADS36* and *MaACS7* promoters. Histochemical detection was operated according to the method described by Jefferson [37].

### Y1H assay

The amplified *MaMADS36* and *MaACS7* promoter regions were fused into the pABAi vector. The Y1H assay was performed according to the method described by Liu *et al*. [22].

### EMSA assay

*MaEIL4* was inserted into the pGEX-4 T-1 vector, and *in vitro* expression and purification yielded GST-*MaEIL4* protein. Then, a 7-bp oligonucleotide probe CTTTGAA harboring EIN3-specific *cis*-elements derived from the *MaMADS36* promoter and a 14-bp oligonucleotide probe GTTTCTTTTTTGGT derived from the *MaACS7* promoter were synthesized. EMSA was accomplished according to the method described by Liu *et al*. [22].

### DLR assay

To evaluate the transcriptional activities of MaEIL4 on the *MaMADS36* promoter and those of MaMADS36 on the *MaACS7* promoter, the promoters were fused into a pGreenII 0800-LUC dual-reporter plasmid. Effector plasmids were generated via the insertion of *MaEIL4* and *MaMADS36* into pGreenII 62-SK. DLR assay was performed according to the method described by Liu *et al*. [22].

### Statistical analyses

All experiments were repeated in triplicate. All data are expressed as the mean ± standard error. The differences among sample means were evaluated by analysis of variance (ANOVA) using a significance level of *P* < 0.05.

### Primers

Table S2 lists all primers used in the present study.

## Supplementary Material

Supplementary_Tables_uhae345

Figure_S1_uhae345

Figure_S2_uhae345

## Data Availability

All experimental data are available and accessible via the main text and/or the supplemental data. Accession number: PRJNA343716.
