# Supplementary figures and images for: MaEIL4-MaMADS36-MaACS7 module transcriptionally regulates ethylene biosynthesis during banana fruit ripening
Source: Hortic Res. 2024 Dec 16;12(3):uhae345. doi: 10.1093/hr/uhae345 (PMC11878764; doi:10.1093/hr/uhae345)

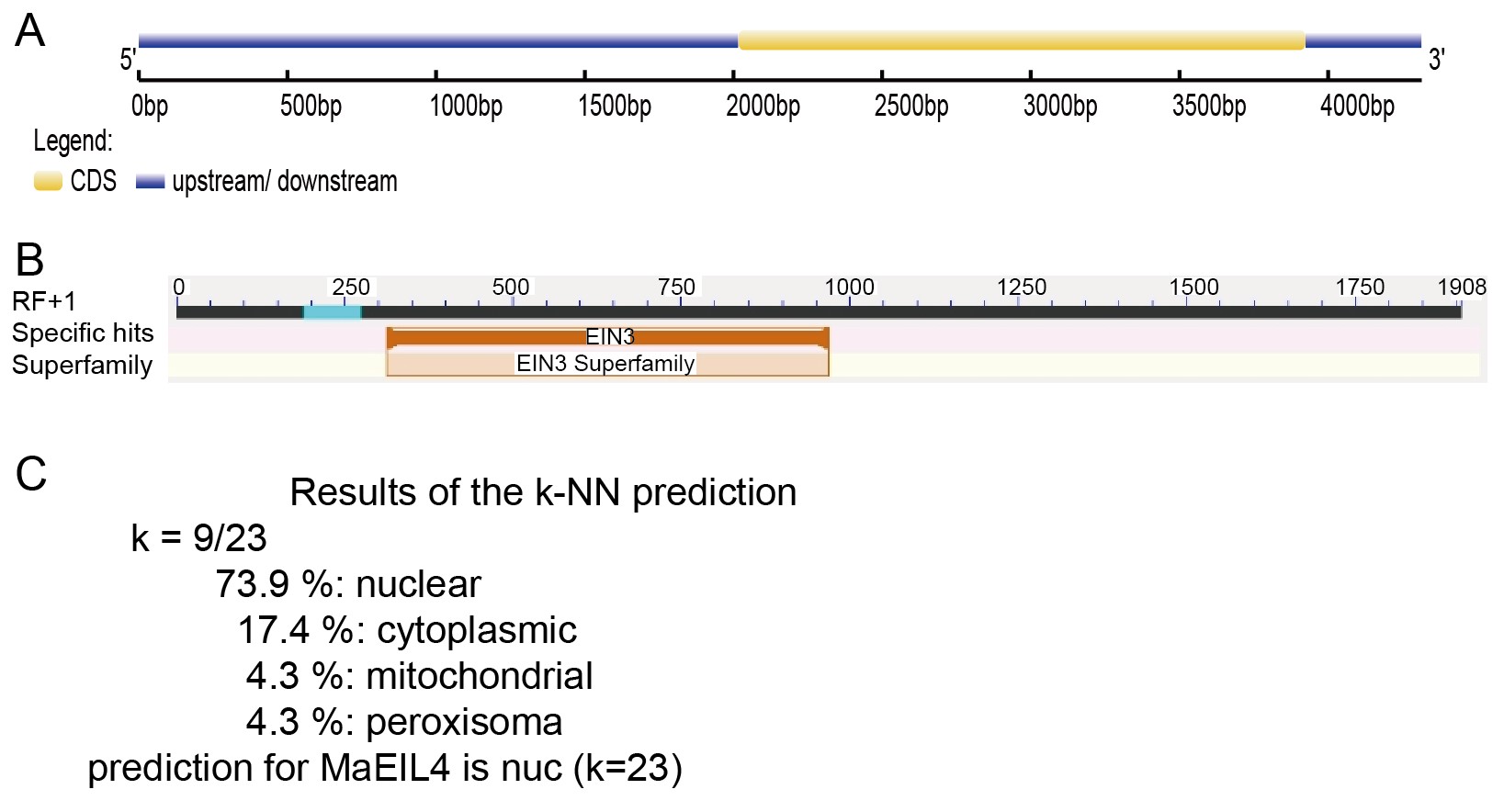

Supplement: Figure_S1_uhae345 [file figure_s1_uhae345.jpeg]

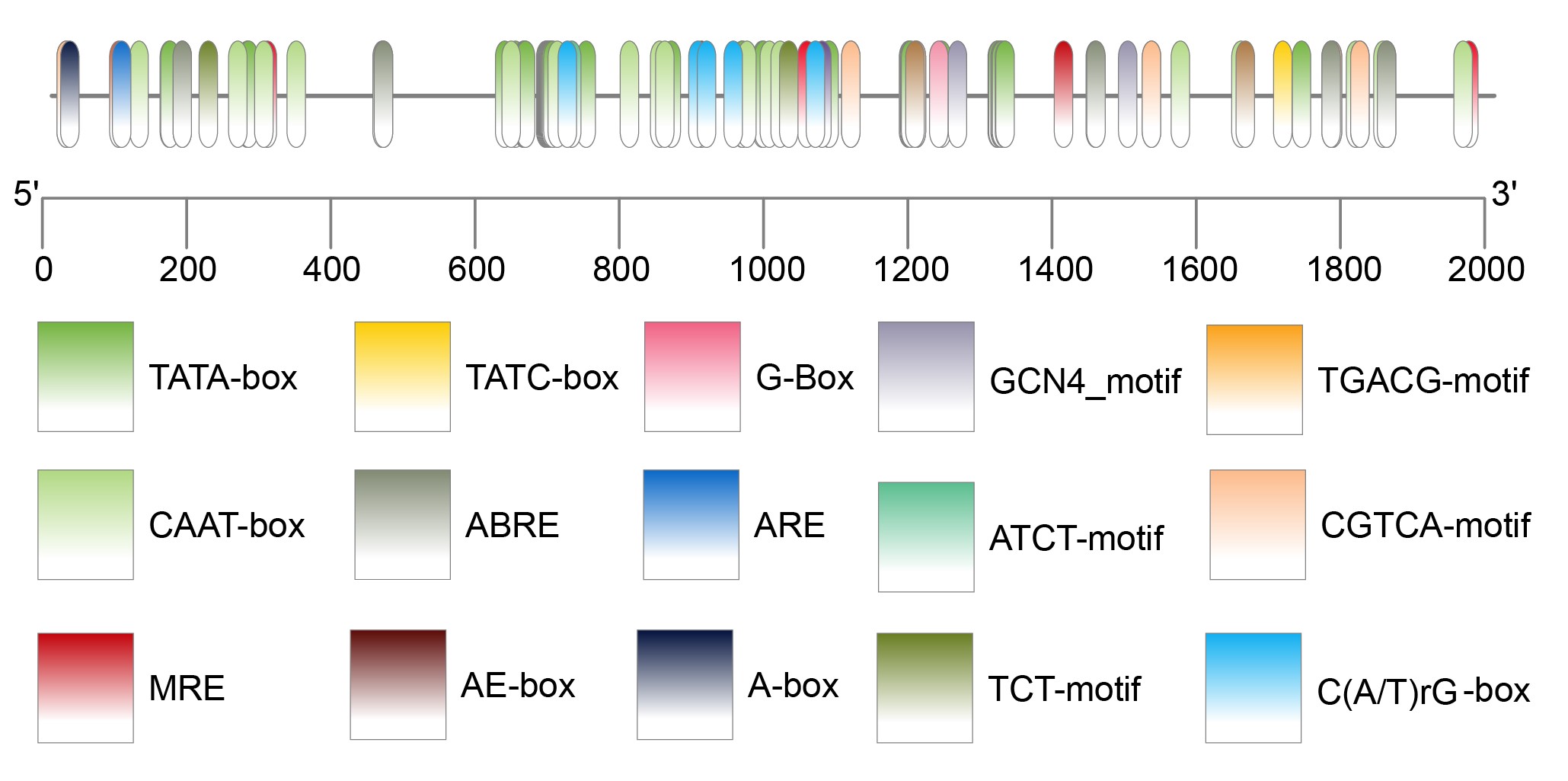

Supplement: Figure_S2_uhae345 [file figure_s2_uhae345.jpeg]
